# Obesity Connected Metabolic Changes in Type 2 Diabetic Patients Treated With Metformin

**DOI:** 10.3389/fphar.2020.616157

**Published:** 2021-02-16

**Authors:** Shereen M. Aleidi, Lina A. Dahabiyeh, Xinyun Gu, Mohammed Al Dubayee, Awad Alshahrani, Hicham Benabdelkamel, Muhammad Mujammami, Liang Li, Ahmad Aljada, Anas M. Abdel Rahman

**Affiliations:** ^1^ Department of Biopharmaceutics and Clinical Pharmacy, School of Pharmacy, The University of Jordan, Amman, Jordan; ^2^ Department of Pharmaceutical Sciences, School of Pharmacy, The University of Jordan, Amman, Jordan; ^3^ Department of Chemistry, University of Alberta, Edmonton, AB, Canada; ^4^ College of Medicine, King Saud Bin Abdulaziz University for Health Sciences, Ministry of National Guard Health Affairs, Riyadh, Saudi Arabia; ^5^ Proteomics Resource Unit, Obesity Research Center, College of Medicine, King Saud University, Riyadh, Saudi Arabia; ^6^ Endocrinology and Diabetes Unit, Department of Medicine, College of Medicine, King Saud University, Riyadh, Saudi Arabia; ^7^ University Diabetes Center, King Saud University Medical City, King Saud University, Riyadh, Saudi Arabia; ^8^ Department of Biochemistry and Molecular Medicine, College of Medicine, Al Faisal University, Riyadh, Saudi Arabia; ^9^ Metabolomics Section, Department of Clinical Genomics, Center for Genomics Medicine, King Faisal Specialist Hospital and Research Centre, Riyadh, Saudi Arabia; ^10^ Department of Chemistry, Memorial University of Newfoundland, St. John’s, NL, Canada

**Keywords:** metabolomics, metformin, obesity, body mass index-BMI, type 2 diabete mellitus, mass spectrometry-LC-MS/MS, amino acid, T2DM

## Abstract

Metformin is widely used in the treatment of Type 2 Diabetes Mellitus (T2DM). However, it is known to have beneficial effects in many other conditions, including obesity and cancer. In this study, we aimed to investigate the metabolic effect of metformin in T2DM and its impact on obesity. A mass spectrometry (MS)-based metabolomics approach was used to analyze samples from two cohorts, including healthy lean and obese control, and lean as well as obese T2DM patients on metformin regimen in the last 6 months. The results show a clear group separation and sample clustering between the study groups due to both T2DM and metformin administration. Seventy-one metabolites were dysregulated in diabetic obese patients (30 up-regulated and 41 down-regulated), and their levels were unchanged with metformin administration. However, 30 metabolites were dysregulated (21 were up-regulated and 9 were down-regulated) and then restored to obese control levels by metformin administration in obese diabetic patients. Furthermore, in obese diabetic patients, the level of 10 metabolites was dysregulated only after metformin administration. Most of these dysregulated metabolites were dipeptides, aliphatic amino acids, nucleic acid derivatives, and urea cycle components. The metabolic pattern of 62 metabolites was persistent, and their levels were affected by neither T2DM nor metformin in obesity. Interestingly, 9 metabolites were significantly dysregulated between lean and obese cohorts due to T2DM and metformin regardless of the obesity status. These include arginine, citrulline, guanidoacetic acid, proline, alanine, taurine, 5-hydroxyindoleacetic acid, and 5-hydroxymethyluracil. Understanding the metabolic alterations taking place upon metformin treatment would shed light on possible molecular targets of metformin, especially in conditions like T2DM and obesity.

## Introduction

T2DM is a chronic progressive metabolic disease characterized by insulin resistance and pancreatic β-cell dysfunction due to uncontrolled hyperglycemia (Chatterjee et al., 2017; Chaudhury et al., 2017; Goyal, 2020). The prevalence of T2DM has been increasing steadily worldwide and is considered a major health concern (Zheng et al., 2018; Goyal, 2020). T2DM is characterized by persistent hyperglycemia, which consequently leads to microvascular and macrovascular complications (Goyal, 2020). Even though the predisposition to T2DM has a strong genetic basis, other factors such as obesity, physical inactivity, and energy-dense diets are significant contributing factors in the development of T2DM (Chatterjee et al., 2017; Zheng et al., 2018).

Obesity is an emerging global chronic health problem attributed to around 44% of T2DM cases (Fried et al., 2013; Leitner et al., 2017), with a close relationship between the two conditions termed “diabesity” (Kurland et al., 2013). Both obesity and T2DM are associated with insulin resistance and disturbances in several metabolic pathways (Leitner et al., 2017; Verma and Hussain, 2017). In addition, obesity results in a significant increase in plasma free fatty acids, glycerol, hormones, pro-inflammatory cytokines, and other factors, which have a pivotal role in developing insulin resistance, the hallmark of T2DM (Kahn et al., 2006). Therefore, understanding the complex metabolic changes underlying the pathophysiology of obesity and T2DM, and how obese individuals develop diabetes are essential goals for disease prevention and therapeutic management.

Metformin is a biguanide derivative oral anti-diabetic drug that has been widely used as a first-line treatment for T2DM with outstanding safety records. This drug is multifunctional, as it acts on multiple tissues and targets different pathways. It inhibits hepatic gluconeogenesis and intestinal glucose absorption and promotes glucose uptake in the liver and skeletal muscles (Viollet et al., 2012). These pharmacological effects are mostly mediated by stimulating the adenosine monophosphate (AMP)-activated protein kinase (AMPK), an enzyme involved in cellular regulation of energy homeostasis, as well as lipid and glucose metabolism (Viollet et al., 2012; Rena et al., 2017). A growing body of evidence suggests that metformin might enhance obesity-induced meta-inflammation by affecting resident immune cells in metabolic organs, including adipose tissue and the liver (Foretz et al., 2019). Randomized controlled clinical trials have shown that metformin is associated with weight loss and waist circumference reduction in obese and overweight diabetic patients compared to placebo (UK Prospective Diabetes Study Group, 1998; Diabetes Prevention Program Research Group, 2012). Additionally, metformin is now considered a bioenergetic drug, as it affects ATP production pathways, particularly glycolysis and oxidative phosphorylation (Andrzejewski et al., 2018). Despite its pleiotropic properties, the precise mechanisms behind metformin mode of action, especially in obese diabetic patients, are still elusive, and various metabolic pathways may be involved.

Metabolomics is a comprehensive analytical approach that identifies changes in metabolites’ levels in a particular biological system in response to specific stimuli and pathogenesis (Liu and Locasale, 2017; Jacob et al., 2018). The advances in informatics tools and analytical instrumentation sensitivity allowed the detection of subtle alterations in biological pathways to provide insight into the mechanisms underlying various physiological conditions and aberrant processes, including diseases (Al-Qahtani et al., 2020). Regarding T2DM and obesity, metabolomics has been widely applied in discovering metabolites biomarkers (Zhang et al., 2017; Urpi-Sarda et al., 2019) and investigating the altered metabolic pathways in both conditions (Park et al., 2015; Bagheri et al., 2018). Additionally, the effect of drugs, including metformin on T2DM and obesity, was examined in mice with diet-induced obesity and T2DM (Tomasova et al., 2019), and in patients with T2DM using the metabolomics approach (Adam et al., 2016). Previous metabolomics studies have investigated the changes in the metabolic profile of either T2DM or obesity alone or studied the effect of metformin selectively on obese or T2DM patients. In this study, mass spectrometry (MS)-based metabolomics approach was used to investigate the metabolic changes associated with long-term metformin administration in human diabetic patients and how this pattern is affected by obesity. Identification of significantly changed metabolites among individual study groups would provide a better understanding of the underlying molecular mechanisms of metformin mode of action in T2DM in the presence and absence of obesity. It would also allow us to demonstrate the obesity-related molecular changes and understand the dynamics of metabolic processes involved in humans with obesity and metformin administration.

## Methods

### Study Population

This study involved two cohorts; obese and lean (non-obese) diabetic patients on metformin from two centers: King Faisal Specialist Hospital and Research Center, and University Diabetes Center, King Saud University Medical City, King Saud University Riyadh, Saudi Arabia, and The University of Jordan Hospital, Amman, Jordan, respectively. The obese cohort included 52 participants divided into 26 obese non-diabetic (control), 16 obese T2DM patients (OT2DM), and 10 obese T2DM patients taking metformin (1,500 mg/day) for at least 6 months (OT2DMMet) (Gu et al., 2020). The lean cohort included 49 participants divided into 25 healthy lean control and 24 lean non-obese T2DM patients taking metformin (1,500 mg/day) for at least 6 months (LT2DMMet). Diabetic participants were diagnosed with T2DM for at least 6 months. Patients with acute or chronic kidney disease, patients with congestive heart failure, smokers, pregnant female patients, females with polycystic ovarian syndrome (PCOS), and patients treated with insulin were excluded from the study. Patients in both cohorts who were taking any other medication rather than metformin were excluded from this study.

### Ethics Statement

All procedures performed in this study involving human participants followed the Declaration of Helsinki's ethical standards and the universal international conference on harmonization-good clinical practice (ICH-GCP) guidelines. This study was reviewed and approved by the Institutional Review Board (IRB) at King Faisal Specialist Hospital and Research Center (KFSHRC) (approval number 2170 013), Riyadh, Saudi Arabia, Institutional Review Board (IRB) at King Saud University (approval number E-19-4234 ) and The University of Jordan Hospital (80/2018/1224) for obese and the non-obese cohorts, respectively. Written informed consent was obtained from all participants.

### Anthropometric Measurements

The body mass index (BMI) for each participant was calculated as body weight (in kilograms) divided by the square of body height (in meters). The BMI was classified into normal (18–24.9 kg/m^2^), overweight (25–29.9 kg/m^2^), obese (30–34.9 kg/m^2^), and morbidly obese (>35 kg/m^2^). Obese and morbid obese participants were included in the obese group, while healthy BMI and overweight participants were included in the lean non-obese group.

### Metabolomic Analysis

Samples from the obese cohort were analyzed using Chemical Isotope Labeling (CIL) (Jacob et al., 2019a). In contrast, the non-obese cohort samples were analyzed using label-free untargeted liquid chromatography-mass spectrometry (LC-MS) (Supplementary Figure S1).

#### CIL-LC-MS Metabolomic Analysis for the Obese Cohort

Serum samples were obtained from three groups; obese non-diabetic (n = 26), obese with T2DM (OT2DM, n = 16), and obese with T2DM on metformin (OT2DMMet, n = 10) and stored at −80°C until further metabolomics analysis. A volume of 15 μL serum was taken out from each sample, and the metabolites were extracted by protein precipitation with 45 μL of methanol. After 2 h of incubation at −20°C, 45 μL of supernatant was taken out and dried down, and then mixed with 25 μL water, 12.5 μL acetonitrile (ACN), 12.5 μL of sodium carbonate/sodium bicarbonate buffer, and 25 μL 12C-dansyl chloride or 13C-dansyl chloride (18 mg/ml in ACN) (Supplementary Figure S1). After vortexing and spinning down, the mixture was incubated at 40°C for 45 min. Then, 5 μL of 250 mM NaOH was added to quench the reaction for 10 min at 40°C. After that, 25 μL of 425 mM formic acid in 1:1 ACN/H_2_O was added to consume excess NaOH.

In order to minimize variations in the total sample amount of individual samples using the step-gradient LC-UV method (Wu and Li, 2012), sample normalization was performed, as described in our recent publication (Dahabiyeh et al., 2020). Each sample was labeled by ^12^C-dansyl chloride and mixed in equal mole amount with a^13^C-dansyl chloride pool sample based on the LC-UV analysis quantification results. The mixtures (2.5 μL containing 0.86 mmol of labeled metabolites) were analyzed by a Thermo Scientific Dionex Ultimate 3000 UHPLC System (Sunnyvale, CA) linked to a Bruker Maxis II quadrupole time-of-flight (Q-TOF) mass spectrometer (Bruker, Billerica, MA) using the same chromatography system and MS parameters described in our previous publication (Dahabiyeh et al., 2020). The Quality control (QC) sample was prepared by mixing the ^12^C- and ^13^C-labeled pooled samples in equal mole amount. A QC injection was performed every 15 LC-MS sample runs. In total, there were 14 QC samples injected and analyzed. Peak pairs with ratio values having > ±25% RSD in the QC samples were filtered out.

The processed samples were analyzed using a Thermo Fisher Scientific Dionex Ultimate 3,000 UHPLC System (Sunnyvale, CA) linked to a Bruker Maxis II quadrupole time-of-flight (Q-TOF) mass spectrometer (Bruker, Billerica, MA). The LC column was an Agilent reversed phase Eclipse plus C18 column (2.1 mm × 10 cm, 1.8 μm particle size, 95 Å pore size), while the mobile phase A was 0.1% (v/v) formic acid in 5% (v/v) ACN, and solvent B was 0.1% (v/v) formic acid in acetonitrile. The LC gradient was: t = 0 min, 20% B; t = 3.5 min, 35% B; t = 18 min, 65% B; t = 21 min, 99% B; t = 34 min, 99% B, with a flow rate of 0.18 ml/min. The MS conditions were as follows: polarity, positive; dry temperature, 230°C; dry gas, 8 L/min; capillary voltage, 4,500 V; nebulizer, 1.0 bar; end plate offset, 500 V; spectra rate, 1.0 Hz.

Bruker Daltonics Data Analysis 4.3 software was first used to convert MS spectra information into cvs files. An in-house developed software, IsoMS (Zhou et al., 2014), was used to process the raw data generated from multiple LC-MS runs by peak picking, peak pairing, and peak-pair filtering to remove redundant peaks. IsoMS files from each injection were aligned together based on the peak’s accurate mass and retention time to generate the aligned file. The missing peak pair information in the aligned file was re-extracted from raw data by Zerofill software (Huan and Li, 2015). The metabolites were positively identified by searching against DnsID Library (www.mycompoundid.org) using retention time and accurate mass (Huan et al., 2015). Putative identification was performed by searching exact mass against MyCompoundID library, which contains 8,021 known human metabolites and 375,809 predicted metabolites (www.mycompoundid.org) (Li et al., 2013).

#### Label-free LC-MS Metabolomic Analysis for the Lean Non-obese Cohort

Serum samples were obtained from two groups; healthy lean (n = 25) and lean with T2DM taking metformin (LT2DMMet, n = 24) and stored at −80°C until metabolomics analysis. To 100 μL of a serum sample, 300 μL of methanol and 10 μL of 2.8 mg/ml DL-o-chlorophenyl alanine internal standard were added, followed by vortex mixing for 30 s. The samples were allowed to stand for 1 h at −20°C, then centrifuged at 12,000 rpm at 4°C for 15 min. A volume of 200 μL of the supernatant was transferred to a vial for LC-MS analysis. Each aliquot (100 µL) was mixed with 300 µL of cold ACN, then vortexed for 30 s before analysis. The mixture was centrifuged for 5min at 15,000 rpm at 4°C The supernatant was dried in a vacuum concentrator. Dry residue was re-dissolved in methanol/water in a ratio of (1:1).

The peak separation of 10 μL of injected sample was performed by Ultimate 3,000 LC combined with Q Exactive MS (Thermo Fisher Scientific, CA, United States) and screened with electrospray ionization (ESI)-MS. The LC system is comprised of an ACQUITY UPLC HSS T3 (100 × 2.1 mm 1.8 μm) with Ultimate 3,000 LC. The mobile phase was composed of solvent A (0.05% formic acid-water) and solvent B (ACN) with a gradient elution (1–16 min, 95–5% A; 16–18 min, 5% A; 18–19 min, 5–95% A; 19–20 min, 95–95% A). The flow rate of the mobile phase was 300 μL/min. The column temperature was maintained at 40°C, and the sample manager temperature was set at 4°C. Mass spectrometry parameters in ESI+ and ESI− modes were kept as follows: heater temperature 300°C; sheath gas flow rate, 45 arb; aux gas flow rate, 15 arb; sweep gas flow rate, 1 arb; spray voltage, 3.0 kV; capillary temperature, 350°C; S-Lens RF level, 30%.

The raw data were acquired and aligned using the compound discoverer software (Thermo Fisher Scientific, United States) based on the m/z value and the ion signals’ retention time. The chemical structures of metabolites were identified according to online databases such as the Human Metabolome database (www.hmdb.ca), Metlin (www.metlin.scripps.edu), and the Mass Bank (www.massbank.jp) using the data of accurate masses and MS/MS fragments.

### Statistical Analysis

MetaboAnalyst version 3.0 (McGill University of Montreal, Montreal, QC, Canada) was used to process the MS data from both cohorts. The raw data was normalized to the sample total median to ensure all samples were normally distributed, log-transformed, and Pareto scaled. A univariate analysis using a volcano plot was performed for each binary comparison to identify significantly differentially expressed metabolites based on a fold-change criterion of greater than 1.5 or less than 0.67 with a false discovery rate (FDR) adjusted *p*-value less than 0.05. The *x*-axis, on the volcano plot, represents the fold change (FC) between two comparison groups, while the *y*-axis represents the *p*-value. Multivariate analysis (partial least square-discriminant analysis (PLS-DA)) was carried out to identify any clustering or separation between the compared data sets.

For statistical analysis among the groups, analysis of variance (ANOVA) using post-hoc Tukey’s analysis method, with multiplicity-adjusted *p*-values, for each comparison was used. This type of analysis seemed best to reduce the probability of making a type 1 error since it supports the testing of pairwise differences due to the unequal group sizes among the experimental and the control groups, as seen in our cohorts. Pearson similarity testing, and Venn diagram presentations to develop Metformine, and diabetes related metabolic patterns were performed using Multiple Professional Profiler (MPP) software (Agilent In, CA).

## Results

### Clinical Characteristics and Demographics of the Study Population

The clinical features and demographic data of the study population, including the two cohorts, are presented in Table 1. The majority of OT2DMMet and LT2DMMet groups were females. While more than half of the OT2DM were males. However, there was no significantly difference in gender between the study groups. In addition, lean cohort individuals had better diabetic parameters, i.e., lower HbA1c% and BMI compared to OT2DMMet in the obese cohort. On the other hand, the overall lipid profile for the LT2DMMet group showed higher values of HDL compared to the obese cohort.

**TABLE 1:** The clinical characteristics and demographic data of the study population.

Cohort	Obese cohort (*n* = 52)						Lean non-obese cohort (*n* = 49)			
	Obese (*n* = 26)		OT2DM (*n* = 16)		OT2DMMet (*n* = 10)		Lean (*n* = 25)		LT2DMMet (*n* = 24)	
	Mean	SD	Mean	SD	Mean	SD	Mean	SD	Mean	SD
Age (y)	34.6	11.9	49.4†	12.1	48.5†	10.9	48.6	6.08	60.3*	8.51
Gender (F/M)	(17/9)	-	(4/12)	-	(7/3)	-	(13/12)	-	(15/9)	-
BMI (kg/m^2^)	38.9	8.90	32.7†	7.61	43.2‡	9.32	25.1	1.95	26.2*	1.76
Glucose (mM)	5.35	0.56	10.18†	4.99	9.89†	3.96	-	-	9.0	5.5
HbA1c (%)	5.3	1.72	8.71†	2.82	8.8†	1.79	-	-	7.11*	1.62
LDL (mmol/L)	3.09	0.79	3.55	0.72	2.50‡	0.76	-	-	2.36	0.70
HDL (mmol/L)	1.20	0.25	1.01	0.21	1.05	0.29	-	-	1.43*	0.38
Trig. (mM)	1.18	0.56	2.01†	1.04	1.55	0.54	-	-	1.39	0.63
Insulin (mU/L)	10.07	6.59	8.07	8.72	7.46	2.84	-	-	-	-
HOMA-IR	2.14	1.56	3.03	2.59	3.21	1.20	-	-	-	-

BMI, body mass index; HbA1c, glycated hemoglobin; HDL, high-density lipoprotein; HOMA-IR, homeostasis model assessment of insulin resistance; LDL, low-density lipoprotein; LT2DMMet, lean type 2 diabetic on metformin; OT2DM, obese type 2 diabetic; OT2DMMet, obese type 2 diabetic on metformin; SD, standard deviation; Trig., Triglycerides. Results are presented as Mean ± S.D.; †*p < 0.05* vs. obese subjects; ‡*p < 0.05* vs. OT2DM; *p < 0.05 vs. OT2DMMet.

### The Overall Metabolomic Analysis and Comparisons of the Obese Cohort Groups

Initially, the metabolic changes associated with metformin administration in patients with T2DM and how these changes would be affected by obesity were identified using the LC-MS approach. The different groups of the obese cohort's metabolic profiles were compared using PLS-DA to examine group clustering and separation. In contrast, the volcano plot was used to investigate the significantly up- or down-regulated metabolites. False discovery rate (FDR)-corrected *p*-values (*y*-axis) and fold change (FC) (*x*-axis) thresholds of 0.05, and 1.5 (0.67), respectively, were applied.

**FIGURE 1:**
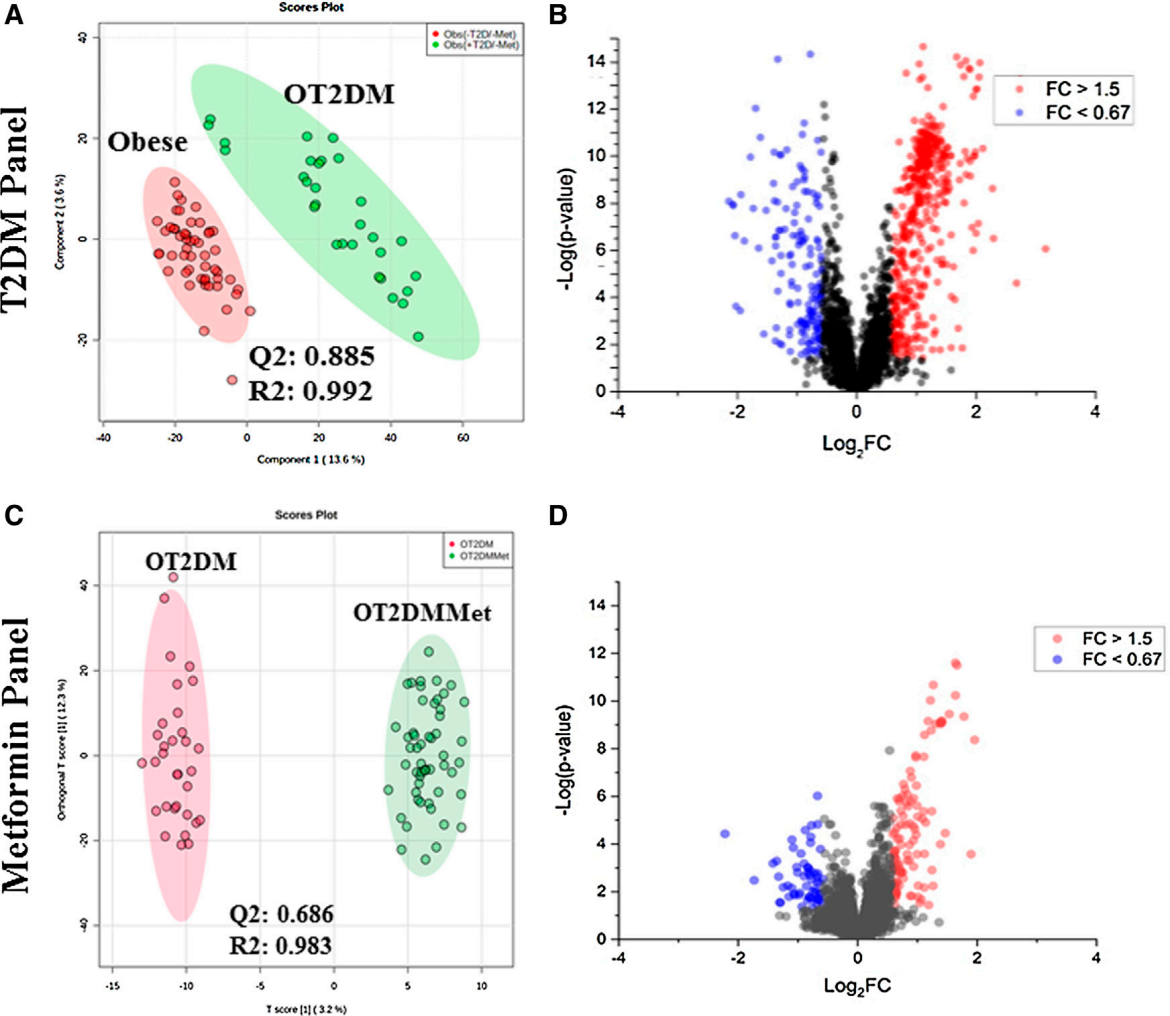
Metabolomics profile of the study population **(A)** Obese vs. obese diabetic patients (OT2DM) metabolomics profile was evaluated using PLS-DA analysis, the clusters of both groups were clearly separated (Q2 = 0.885, R = 0.992) **(B)** Volcano plot of obese vs. OT2DM groups shows the significantly changed metabolites. 459 metabolities were up-regulated and 166 were down-regulated in OT2DM group with fold change and FDR adjusted *p*-value at the cut-off 1.5 (or 0.67), and (0.038, respectively **(C)** Obese diabetic (OT2DM) vs. Obese diabetic taking metformin (OT2DMMet) metabolomic profile was evaluated using PLS-DA analysis, the clusters of both groups were clearly separated (Q2 = 0.686, R2 = 0.983) **(D)** Volcano plot of OT2DM vs. OT2DMMet group shows levels of 107 metabolites were significantly different as of metformin, 78 were upregulated and 29 were down-regulated in obese diabetic patients taking metformin. The fold change and FDR adjusted *p*-value at the cut-off 1.5 (or 0.67) and 0.05, respectively.

The PLS-DA score plot of T2DM was built by comparing obese vs. OT2DM groups from the obese cohort (T2DM panel, Figure 1A). The PLS-DA score plot revealed a clear separation and grouping between these two groups (Q2 = 0.885, R2 = 0.992), reflecting that T2DM itself has a significant effect on the dynamic of metabolic processes in obese patients. The volcano plot (T2DM panel, Figure 1B) showed a significant change in the levels of several metabolites, of which 459 metabolites were up-regulated, and 166 were down-regulated in the OT2DM group (identified and unidentified).

Metformin metabolomic pattern in obese diabetic patients was examined through a PLS-DA comparison between OT2DM vs. OT2DMMet groups from the obese cohort. As shown in the metformin panel of Figure 1C, evident separation of the compared groups was noted in the PLS-DA score plot (Q2 = 0.686, R2 = 0.983), indicating that metformin administration as well has a significant effect on the dynamic of metabolic processes in obese diabetic patients. A total of 107 metabolites (identified and unidentified) were significantly dysregulated of which 78 were up-regulated, and 29 were downregulated in obese diabetic patients on metformin as shown in metformin panel, volcano plot, Figure 1D.

### T2DM Metabolic Changes in Obesity

**FIGURE 2:**
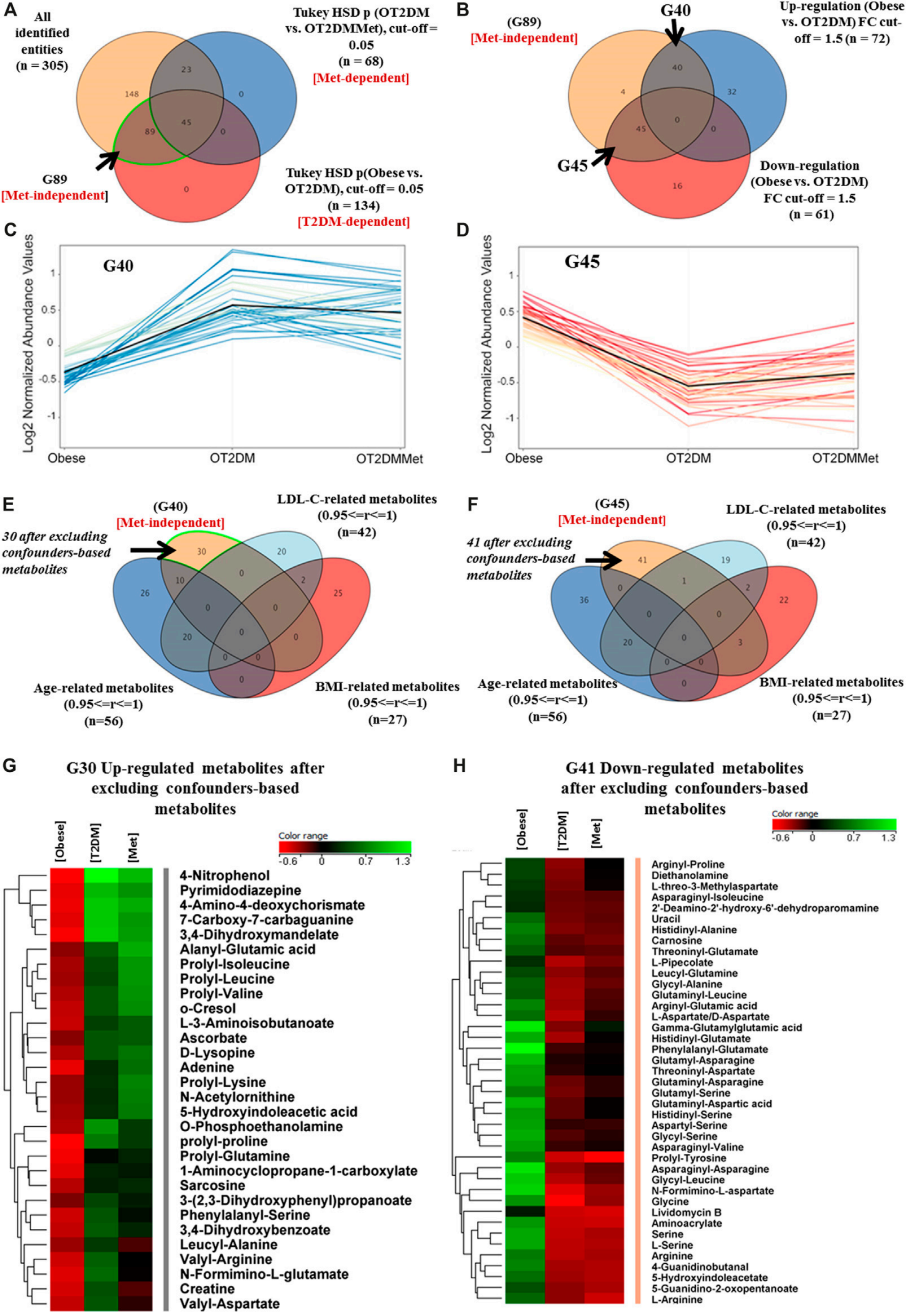
Metabolic pattern of T2DM effect on metabolites that are irresponsive to metformin administration in obesity **(A)** Venn diagram illustrates the extraction of 89 metabolite (G89) that were significantly dysregulated due to diabetes but not metformin effect (metformin-independent) **(B)** Venn diagram shows the breakdown of the G89 (Met-independent) after applying the fold change cutoff 1.5, where 40 and 45 metabolites were up and downregulated in diabetes, respectively **(C)** 40 (G40) metabolites were up regulated as of T2DM and their levels were unchanged after metformin administration **(D)** 45 (G45) metabolites were down regulated as of T2DM and their levels were unchanged after metformin administration **(E, F)** Venn diagrams demonstrate the exclusion of the confounders-based metabolites from G40 and G45, which were reduced to G30 and G41, respectively **(G, H)** Heat maps of the identified features (G30 and G41) that were either upregulated or down regulated, respectively, after excluding the confounder-based metabolites as of T2DM effect in obesity.

A panel of 305 features was commonly identified in the binary comparisons between the study groups. The metabolic pattern associated with T2DM in obesity was built based on the identified features extracted from the comparison between obese vs. OT2DM groups (T2DM-dependent, Figure 2A). Metformin responsive features (n = 68) were identified from the comparison between OT2DM vs. OT2DMMet groups (Met-dependent, Figure 2A), and were excluded from the analysis. Considering T2DM metabolic panel (n = 134), the features that were irresponsive to metformin (n = 89), were identified (Metformin-independent, Figure 2A). These metformin-independent metabolites were changed as an effect of T2DM but not metformin treatment. Their fold change was analyzed (cutoff 1.5), as shown in Figure 2B, and 40 and 45 metabolites were found to be up- and down-regulated, respectively, in T2DM compared to obese groups (Figures 2C,D). Metabolite levels in human serum are highly sensitive to certain parameters such as age, BMI, and low-density lipoprotein-cholesterol (LDL-C) (Jacob et al., 2019b). In this study, age, BMI, and LDL-C were considered confounders, and the values of these confounders were integrated into the metabolomics dataset. After excluding the confounders-based metabolites from the up- and down-regulated metabolites, 30 and 41 metabolites resulted, respectively, as demonstrated in Figures 2E,F. These dysregulated metabolites' identity as an effect of T2DM in obesity and their levels in different study groups are shown in Figures 2G,H.

### Metabolic Changes of Metformin Administration in Obese Diabetic Patients

**FIGURE 3:**
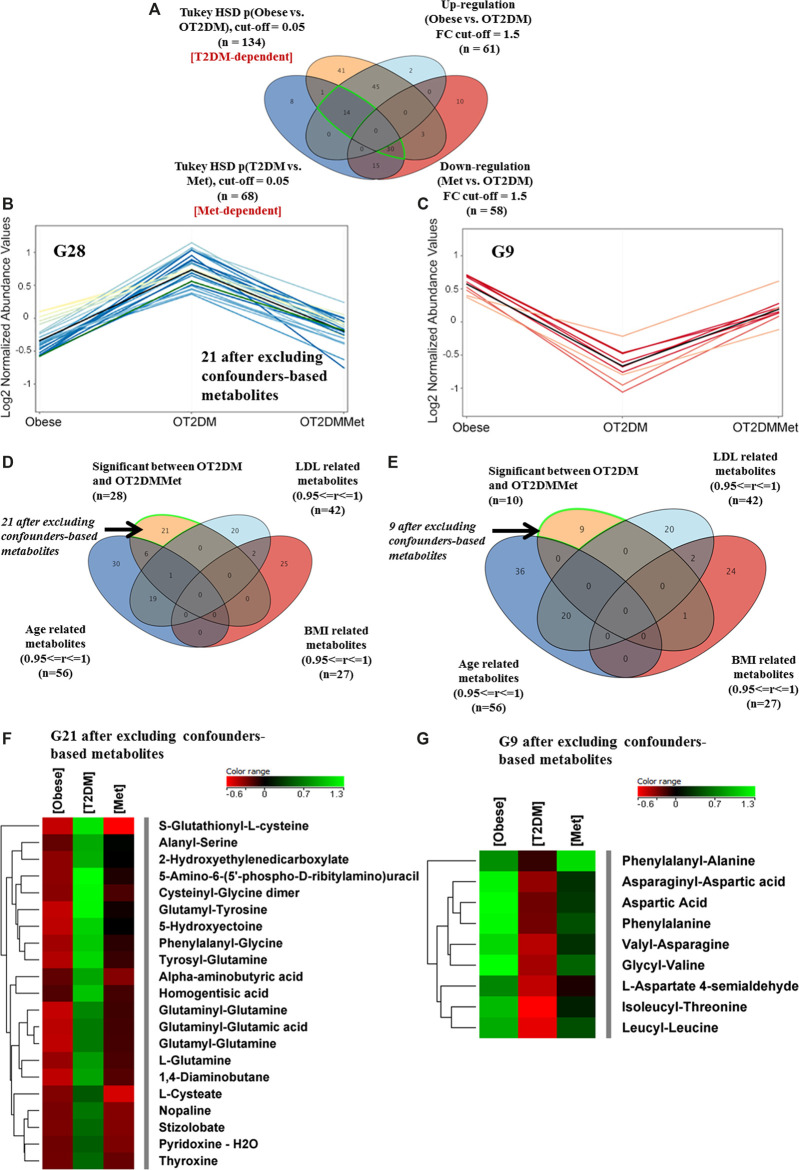
Metabolic changes of metformin administration in obese diabetic patients **(A)** Venn diagram illustrates the filtration of the significant metabolites for T2DM (G134) (Obese vs. OT2DM groups), and metformin (G68) (OT2DM vs. OT2DMMet groups) based on the fold change trend (cutoff >1.5, and <0.67) **(B)** 28 metabolites have been upregulated as of diabetes and returned to obese comparable levels after metformin administration **(C)** Levels of another 9 metabolites were downregulated in T2DM and returned to obese comparable levels after metformin administration **(D**,**E)** Venn diagrams demonstrate the exclusion of the confounders-based metabolites from G28 and G10, which were reduced to G21 and G9, respectively **(F, G)** Heat maps of the identified features (G21 and G9) that were-upregulated and down-regulated, respectively, after excluding the confounder-based metabolites as of T2DM effect in obesity and return to initial condition after metformin administration.

The effect of metformin administration on metabolites in obese diabetic patients was investigated based on comparing OT2DM vs. OT2DMMet groups from the obese cohort. The T2DM metabolic pattern (n = 134) that has been generated from the ANOVA comparison between the obese and OT2DM (Figure 2A) was overlapped with metformin responsive features (n = 68) (Metformin-dependent panel, Figure 3A). After fold change analysis (cutoff >1.5, and <0.67), 30 and 14 metabolites were up- and down-regulated due to metformin administration in T2DM, respectively. Further filtration, considering identified features which were affected by T2DM and metformin (Supplementary Figure S2), showed that only 28 (out of 30) and 9 (out of 14) metabolites were up- and down-regulated in OT2DM and then returned to obese group comparable levels by metformin administration in the obese diabetic patients (Figures 3B,C). Additionally, the confounders-based metabolites were overlapped and removed from G30 and G14, to result in 21 and 9 metabolites that were up- and down-regulated due to metformin administration in OT2DM, respectively (Figures 3D,E). The identity of these dysregulated metabolites (G21 and G9) and their levels in different study groups are presented in a heatmap format (Figures 3F,G).

**FIGURE 4:**
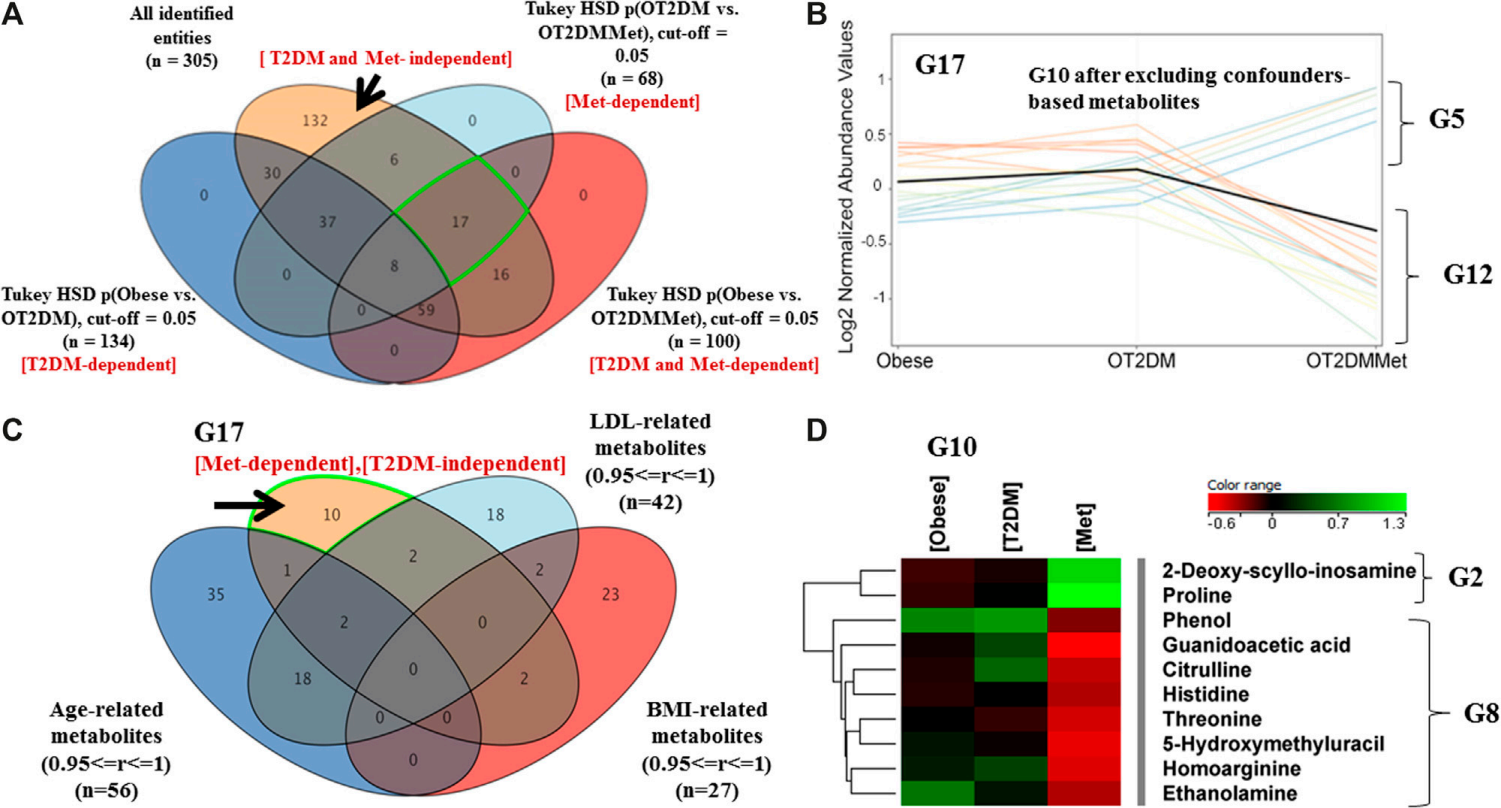
Metabolic pattern of metformin administration effect on metabolites that are not changed with T2DM in obesity **(A)** Venn diagram illustrates the extraction of 17 metabolite that were non-significant as of T2DM (T2DM-independent), as well as 132 metabolites that are irrespective neither to T2DM nor to metformin administration (T2DM and Met-independent) **(B)** Five metabolites (G5) wereup regulated, and 12 (G12) were down regulated as of metformin administration only and their levels were unchanged as of T2DM (FC cutoff is 1.5) **(C)** Venn diagram demonstrates the exclusion of the confounders-based metabolites from G17 **(D)** Heat map for the metabolites that were either upregulated (G2) or down regulated (G8) after excluding the confounder-based metabolites as of metformin administration in obese diabetic patients.

An investigation on metformin administration alone, without the effect of T2DM, on the obesity metabolic pattern, was also considered. As shown in Figure 4A, 17 metabolites were statistically significant due to metformin administration in obese diabetic patients. The metabolic pattern of these metabolites considering fold change trend, cutoff >1.5, and <0.67, indicated that 5 metabolites were up-regulated, while 12 metabolites were down-regulated in the OT2DMMet group, (Figure 4B). After excluding the confounders-based metabolites, the identified metabolites were reduced to have only 10 total metabolites responsive to metformin (Figure 4C). Among these metabolites, 2 (out of 5) and 8 (out of 12) metabolites were up- and down-regulated as an effect of metformin in obese diabetic patients (Figure 4D).

### Metabolites That Are Irresponsive Neither to T2DM nor to Metformin Administration in Obese Diabetic Patients

The metabolites which neither perturbed with T2DM nor metformin in the obese cohort were considered in this analysis. As shown in Figure 4A, comparing groups of obese vs. OT2DM and groups of OT2DM verses OT2DMMet, resulted in extracting 132 metabolites, which were uncommon between T2DM and metformin (T2DM and Met-independent). These 132 metabolites showed a persistent metabolic pattern as an effect of both T2DM and metformin administration. After applying the fold change filtration (cutoff >1.5, and <0.67), only 113 metabolites were found as independent entirely on the effect of either T2DM or metformin administration (Supplementary Figure S3A). Considering the confounders-based metabolites, 113 metabolites were reduced to have only 62 (Supplementary Figure S3B).

### Metabolites That Have Been Affected by T2DM and Metformin Regardless of Obesity Status

**FIGURE 5:**
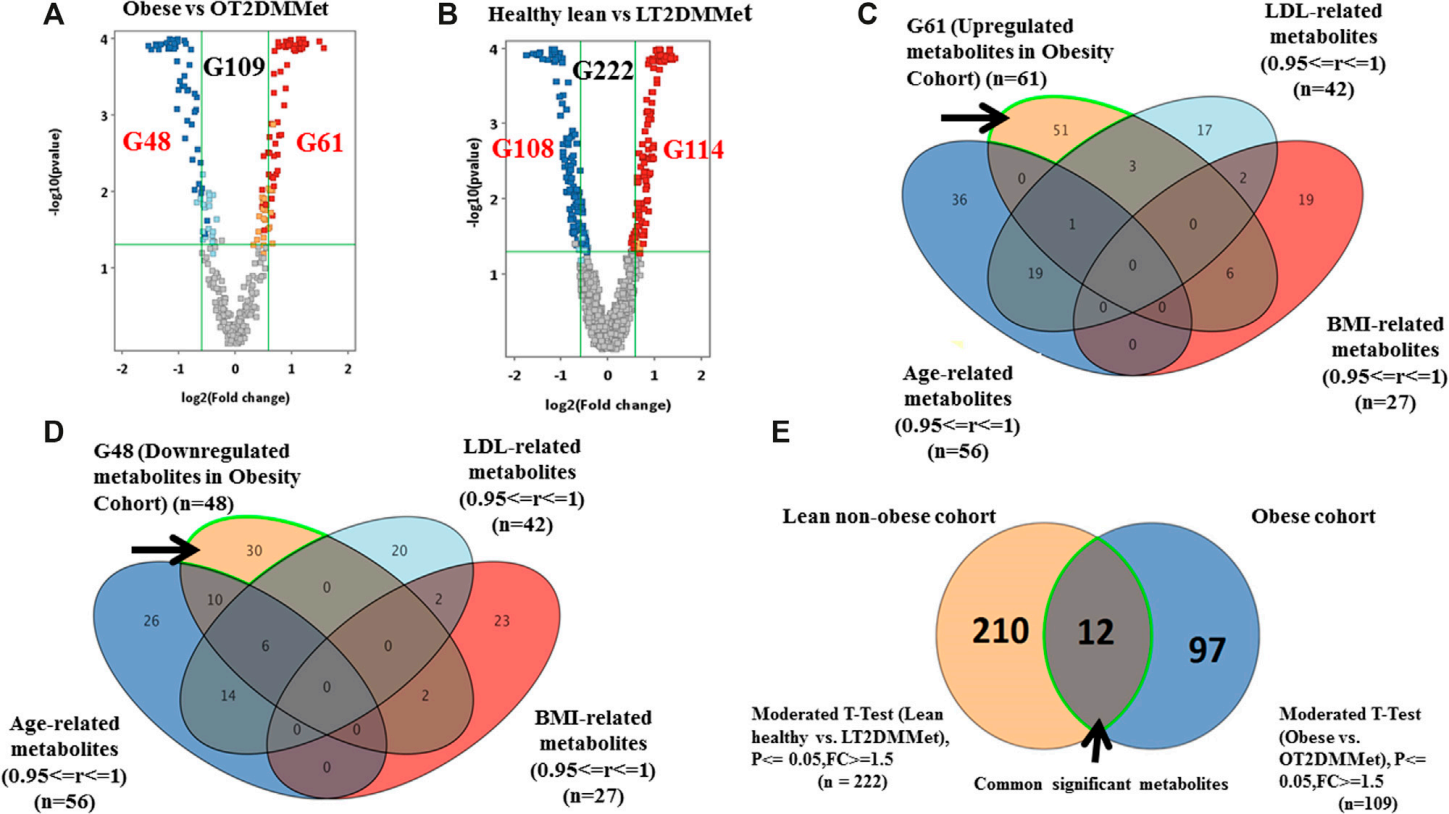
Significant metabolites that are common between obese and lean cohorts **(A)** Volcano plot pf obese vs. OT2DMMet groups from the obese cohort shows the significantly changed metabolites. A total of 109 metabolites (G109) were significant, (G61) down-regulated after metformin administration in obese diabetic patients compared to obese non-diabetic participants, the fold change and FDR-adjusted *p*-value at the cutoffs were 1.5 and <0.05, respectively **(B)** Volcano plot of lean healthy vs. LT2DMMet groups from the lean cohort shows that a total of 222 metabolites (G222) were significant between compared groups, where (G114) were up regulated and (G108) were down regulated after metformin administration in lean non-obese diabetic patients compared to healthy lean, with fold change and FDR adjusted *p*-value at the cut-off 1.5 and 0.05, respectively, **(C**, **D)** Venn diagrams demonstrate the exclusion of the confounders-based metabolites from G61 and G48, where the metabolites were reduced to G51 and G30, respectively **(E)** Venn diagram shows that 12 metabolites are common between obese and lean non-obese cohort using moderate *t*-Test.

As mentioned in the previous sections, the metabolic pattern of metformin administration was investigated in the obese diabetic cohort considering the BMI as a confounding factor. However, to investigate the effect of both metformin and T2DM on the metabolite levels regardless of other obesity features (i.e., insulin resistance), comparisons were carried out considering groups from obese and lean non-obese cohorts. Volcano plot analysis between obese vs. OT2DMMet groups resulted in 109 significantly dysregulated metabolites. Among these metabolites, 61 were up-regulated, and 48 were down-regulated in OT2DMMet compared to the obese group (Figure 5A). Additionally, another volcano plot analysis between healthy lean vs. lean non-obese diabetic on metformin (LT2DMMet) groups from the lean non-obese cohort revealed that the levels of 222 metabolites were significantly altered. Among these metabolites, 114 were up-regulated, and 108 were down-regulated in LT2DMMet compared to the healthy lean group (Figure 5B).

Considering the confounders-based metabolites, the dysregulated metabolites in OT2DMMet compared to the obese group were reduced to (51 up-regulated (out of 61) and 30 down-regulated (out of 48)) (Figures 5C,D). Furthermore, overlapping G109 from the obese cohort with G222 from the lean non-obese cohort, resulted in 12 metabolites, which were highlighted in the venn diagram as common and significant metabolites between the two cohorts using moderate *t*-test (*p*-value <0.05, Fold change≥ 1.5 or =<0.67). After considering the confounders-based metabolites, 9 metabolites remained out of 12. These 9 metabolites were affected by T2DM and metformin regardless obesity status. The identity of these metabolites and their regulation pattern due to metformin administration and T2DM effects in the two cohorts are presented in Table 2.

**TABLE 2:** Common metabolites between obese and lean models and their metabolic pattern as an effect of metformin and T2DM.

Metabolite ID	HMDB ID	Obese cohort FC^¥^ (*p*-value)	Lean cohort FC^¥^ (*p*-value)
L-Arginine	HMDB0000517	↓2.08 (6.36E-5)	↓1.65 (9.89E-3)
Guanidoacetic acid	HMDB0000128	↓2.39 (2.61E-8)	↓2.18 (2.21E-5)
5-Hydroxyindoleacetic acid	HMDB0000763	↑2.0 (2.47E-4)	↓2.07 (9.22E 05)
L-Proline	HMDB0000162	↑1.98 (1.05E-5)	↑1.63 (1.16E-02)
Taurine	HMDB0000251	↓1.62 (1.5E-4)	↓1.89 (7.67E-4)
L-Alanine	HMDB0000161	↑1.67 (0.00045)	↑1.85 (1.26E-3)
Citrulline	HMDB0000904	↓1.65 (0.004)	↓2.05 (1.15E-4)
5-Hydroxymethyluracil	HMDB0000469	↓2.25 (3.31E-6)	↓2.35 (1.71E-6)
Salicylic acid	HMDB0001895	↑1.52 ()	↑1.61 (1.46E-2)

FC: Fold change ↑: up-regulated or ↓: down-regulated in the metformin-treated group compared to diabetic untreated in both cohorts.¥ Regulation in metformin treated group compared to untreated in both cohorts.

## Discussion

This study aimed to use the LC-MS-based metabolomics approach to investigate the metabolic pattern of long-term metformin administration in diabetic patients and how this pattern becomes affected in obesity, particularly since most diabetic patients on metformin are obese. Our results show a clear group separation and sample clustering between the study groups due to T2DM and metformin administration. This observation represents the significant effect of these combined conditions on the dynamic of metabolic processes in obesity. Investigating the T2DM metabolic pattern in obesity revealed that 30 and 41 metabolites were up- and down-regulated, respectively, in T2DM. The levels of these total 71 metabolites were unchanged with metformin intake, indicating that they are probably related to dysregulated pathways associated with T2DM or to its complications. The effects of confounder parameters (i.e., age, BMI, and LDL-C), were excluded; therefore, these dysregulated metabolites are mostly independent of body weight. However, they might be influenced by other obesity-related features such as insulin resistance, hormones, and pro-inflammatory cytokines. Most of the up-regulated metabolites, as an effect of T2DM, were dipeptides containing branched amino acids (BCAAs) in their structure (i.e., prolyl-leucine, prolyl-isoleucine, prolyl-valine, leucyl-alanine, and valyl-arginine). Several previous studies have reported a strong positive correlation between the levels of a number of amino acids, including BCAAs and obesity-associated insulin resistance and the risk of developing T2DM (Newgard et al., 2009; Wang et al., 2011; Chen et al., 2016; Guasch-Ferré et al., 2016; Ho et al., 2016; Chen et al., 2019). Amino acids and their metabolites are an important regulator of insulin secretion and sensitivity (Newgard, 2012). They also play a crucial role in glucose metabolism, particularly gluconeogenesis, and tricarboxylic acid (TCA) cycle by regulating the substrate availability (Menge et al., 2010). Several possible mechanisms might underlie the dysregulation of these amino acids' levels and their metabolites in obese diabetic patients, including changes in gene expression of the enzymes involved in their catabolic pathways or protein synthesis and insulin signaling pathways (Roberts et al., 2014; Guasch-Ferré et al., 2016).

In this study, aliphatic amino acids such as arginine, serine, and glycine were downregulated in T2DM. Different metabolomic investigations have consistently reported a negative correlation between the levels of some aliphatic amino acids and insulin sensitivity and T2DM (Menge et al., 2010; Ha et al., 2012; Wang-Sattler et al., 2012; Floegel et al., 2013b). However, so far, there is no exact mechanism explaining this reverse correlation. One proposed mechanism is related to insulin resistance. It has been suggested that insulin resistance might reduce glycine level by inducing δ-aminolevulinic acid synthase 1 (ALAS1) that produces 5-aminolevulinic acid from glycine (Wang-Sattler et al., 2012; Roberts et al., 2014). Another proposed mechanism is related to oxidative stress associated with diabetes, which increases the demand for glutathione and decreases circulating glycine, as it acts as a precursor of glutathione (Ferrannini et al., 2013; Roberts et al., 2014).

Moreover, our results show that serine is down-regulated in T2DM, indicating a possible alteration in its metabolic pathway. Serine is a non-essential amino acid synthesized from 3-phosphoglycerate (3PGA), an intermediate of the glycolytic pathway (Ma et al., 2018). The final production of serine amino acid requires glutamic acid, which is reduced in T2DM patients (Ma et al., 2018). In addition, serine could be synthesized from glycine, which is another non-essential amino acid (Haufroid and Wouters, 2019), indicating that these amino acids' metabolic pathways are connected.

Among the up-regulated metabolites in T2DM is creatine, which is produced from arginine. A non-enzymatic breakdown of creatine results in creatinine formation, a well-known measure for kidney function (Post et al., 2019). Therefore, the observed down-regulated arginine levels in T2DM might be explained by the high demand for its conversion to creatine.

The results indicate that 30 metabolites were dysregulated in obese diabetic patients (21 were up-regulated, and 9 were downregulated) and restored to obese control levels after metformin administration. Two possible explanations for such a pattern of these metabolites. First, these identified metabolites are mostly sensitive to metabolic pathways related to both T2DM and metformin since their levels were changed in the two conditions, but in a reverse manner. Secondly, these metabolites might be altered in the case of T2DM, and then metformin administration adjusts the affected pathways in T2DM. For example, evidence linked the increase in glutamate and glutamate-to-glutamine ratio with insulin resistance and the incidence of T2DM (Cheng et al., 2012; Roberts et al., 2014; Arneth et al., 2019; Chen et al., 2019). However, others did not report an association with diabetes (Floegel et al., 2013a). In this study, several glutamine-containing dipeptides (i.e., Glutaminyl-Glutamine, Glutaminyl-Glutamic acid, Glutamyl-Glutamine, Tyrosyl-Glutamine, Glutamyl-Lysine, Glutamyl-Tyrosine) as well as glutathionyl-L-cysteine were significantly up-regulated in T2DM, and then returned to obese comparable levels after metformin administration. This might suggest an effect of metformin on the γ-glutamyl cycle. Existing evidence suggests that metformin affects glutamine metabolism. *In vitro* studies have shown that metformin has an inhibitory effect on glutaminase (GLS) activity in tumor cells (Ampuero et al., 2012; Saladini et al., 2019). Glutaminase enzyme mediates glutamine deamination and subsequent glutamate production, converted to α-ketoglutarate, an intermediate of the TCA cycle, and thus provides an alternative energy source to glucose in cells (Roberts et al., 2014). However, there is no evidence on metformin's effect on glutamine metabolism or the γ-glutamyl cycle in human metabolic disorders. Taken together, dysregulation of glutamine derivative metabolites as an effect of metformin in obese diabetic patients needs further investigation since the present evidence was from *in vitro* studies on cancer cells.

Furthermore, in obese T2DM patients, the level of 10 metabolites was dysregulated only after metformin administration. These metabolites are most likely related to the metformin mode of action or its side effects in the body. It has been suggested that metformin can create metabolic stress in cells by inhibiting the respiratory chain complex I of the mitochondria and partially by inhibiting NADH dehydrogenase (Madiraju et al., 2014; Rena et al., 2017; Vial et al., 2019). Consequently, this would lead to some metabolic disruptions, including decreased NADH oxidation, reduction in TCA flux, and levels of its metabolites (Andrzejewski et al., 2018; Vial et al., 2019). Interestingly, none of the identified downregulated metabolites in the metformin-treated obese T2DM group was a direct TCA metabolite.

On the other hand, among the down-regulated metabolites in the metformin-treated obese T2DM group are those of the urea cycle, i.e., citrulline, and homoarginine, which is the methylene homologue of arginine. These findings imply that metformin might influence amino acids’ metabolic pathways involved in urea production and ammonia elimination in obesity. In line with our findings, previous metabolomics studies indicated that metformin administration is associated with reduced serum levels of urea cycle metabolites in humans with and without diabetes (Irving et al., 2015; Brandmaier et al., 2015; Adam et al., 2016; Rotroff et al., 2016). However, the exact explanation for decreasing these metabolites after metformin administration remains elusive (Farriol et al., 2001).

The metabolic pattern of 62 metabolites was persistent and the levels of these metabolites were affected by neither T2DM nor metformin in obesity. These metabolites are most probably independent entirely from the pathways involved in both T2DM and metformin. They are mostly related to the presence of other factors, such as obesity and insulin resistance. In another metabolic analysis, we investigated the specific metabolic effect of insulin resistance in diabetic patients, and we have detected several dysregulated metabolites ( (Gu et al., 2020).

This study also revealed that 9 metabolites were significantly dysregulated between lean and obese cohorts. The metabolic pattern of these metabolites was changed in the metformin-treated group compared to the untreated group regardless obesity status. These identified metabolites are mostly independent on BMI and other obesity features, i.e., insulin resistance, as they are common between the two cohorts. The regulation of these 9 metabolites levels was the same in both cohorts except for 5-hydroxyindoleacetic acid. It was up-regulated in ODMMet and down-regulated in LDMMet compared to untreated groups in both cohorts. The exact explanation for this pattern is unknown, but several factors might be involved. 5-hydroxyindoleacetic acid is the primary metabolite of serotonin, and its serum or urinary levels reflect the serotonin levels in the body. It has been indicated that the serotonin metabolism pathway might be altered in T2DM and obesity (Matsuoka et al., 2016; Martin et al., 2017). Plasma levels of 5-hydroxyindoleacetic acid were increased in diabetic patients who had lower plasma levels of tryptophan, indicating serotonin's catabolism to 5-hydroxyindoleacetic acid is increased when the plasma tryptophan level is low in diabetes (Matsuoka et al., 2016).

Regarding the effect of metformin on the serotonin's metabolism pathway, it has been found that metformin interacts with proteins that are involved in the intestinal disposition of serotonin and histamine (Yee et al., 2015). Metformin inhibits serotonin and histamine uptake in the intestine through interaction with amine transporters (organic cation transporters (OCT1), OCT3, and serotonin transporter (SERT)) in a dose-dependent manner (Yee et al., 2015). Therefore, it has been suggested that metformin modulates the intestinal or systemic serotonin levels, which contributes to the gastrointestinal side effect of metformin (Yee et al., 2015). Overall, obesity status appeared to modify the metabolic effect of metformin and T2DM on the serotonin's catabolism pathway.

In the current study, L-arginine and guanidoacetic acid were among the down-regulated metabolites in metformin-treated diabetic groups in both obese and lean cohorts. Guanidoacetic acid is involved in numerous amino acids’ metabolic pathways, including; arginine, glycine, serine, threonine, and proline. Limited studies have investigated the metabolic pattern of arginine and guanidoacetic acid regarding conditions such as metformin administration, T2DM, or obesity. In a cohort of healthy subjects, elevated serum level of guanidoacetic acid was associated with hyperinsulinemia, higher total homocysteine, and higher body fat percentage (Ostojic et al., 2018), which all are features of obesity and T2DM. Therefore, future studies are required to investigate metformin's effect on the pathways that involve arginine and guanidoacetic acid in obesity.

In addition, the levels of taurine, citrulline, and 5-hydroxymethyluracil (HMU) were also decreased, while levels of L-proline, L-alanine, and salicylic acid were increased in the metformin-treated diabetic group in both obese and lean cohorts. The existing evidence on the effect of metformin on the amino acids' levels in T2DM is conflicting. Some studies have reported an increase in the levels of BCAAs and aromatic amino acids (AAAs) after metformin administration in subjects with a high risk of developing T2DM (Walford et al., 2013). Moreover, the effect of metformin on the BCAAs and AAAs levels was found to be more pronounced in insulin-resistant subjects (Walford et al., 2013). Interestingly, metabolomic studies of obesity and metabolic syndrome have shown an increase in alanine levels (Libert et al., 2018). Furthermore, the metabolic signature in obese subjects is marked with a significant increase in the serum levels of amino acids, including alanine (Rangel-Huerta et al., 2019). Metformin is believed to inhibit gluconeogenesis in the liver (Rena et al., 2017). Subsequently, metformin intake would increase gluconeogenic substrate such as alanine, which is in concordance with our findings. Recent metabolomic analysis of mouse embryonic fibroblast (MEF) cells treated with metformin showed that metformin significantly reduced the TCA and affected several amino acids' metabolism, bypassing AMPK (Yan et al., 2019). Of these amino acids are up-regulated hypotaurine (precursor of taurine) and downregulated proline (Yan et al., 2019), inconsistent with our findings in humans treated with metformin.

Finally, in this study, serum samples from the obese and the lean non-obese cohorts were analyzed using CIL-LC-MS and LC-MS, respectively. Although the use of the two platforms in the analysis may appear to be a limitation, the results showed the detection of significant meaningful changes in several metabolites' levels. Moreover, recruiting lean T2DM patients was challenging in this study; since most of T2DM patients are overweight or obese. Despite these limitations, considering different groups from the two cohorts, including obese, healthy lean, obese and lean diabetic with and without metformin intake, enabled us to carry out several comparisons to extract the significant changes in the identified metabolites' patterns.

## Conclusion

Understanding the metabolic alterations taking place upon metformin treatment would shed light on possible molecular targets of metformin, especially in conditions like T2DM and obesity. These conditions by themselves contribute to the perturbation of several metabolic pathways in the body. The present study has revealed significant changes in different metabolites; some were specific for metformin and T2DM regardless of obesity. This data would facilitate metabolomic analysis to establish a metformin sensitivity profile in humans and consequently identify patients who would be most sensitive and responsive to metformin treatment regardless of obesity.

## Data Availability

The original contributions presented in the study are included in the article/Supplementary Material, further inquiries can be directed to the corresponding authors.

## References

[B1] Adam J. Brandmaier S. Leonhardt J. Scheerer M. F. Mohney R. P. Xu T. (2016). Metformin effect on nontargeted metabolite profiles in patients with type 2 diabetes and in multiple murine tissues. Diabetes 65, 3776–3785. 10.2337/db16-0512 27621107

[B2] Al-Qahtani W. Abdel Jabar M. Masood A. Jacob M. Nizami I. Dasouki M. (2020). Dried blood spot-based metabolomic profiling in adults with cystic fibrosis. J. Proteome Res. 19, 2346–2357. 10.1021/acs.jproteome.0c00031 32312052

[B3] Ampuero J. Ranchal I. Nuñez D. Díaz-Herrero M. M. Maraver M. del Campo J. A. (2012). Metformin inhibits glutaminase activity and protects against hepatic encephalopathy. PloS One 7, e49279. 10.1371/journal.pone.0049279 23166628 PMC3499552

[B4] Andrzejewski S. Siegel P. M. St-Pierre J. (2018). Metabolic profiles associated with metformin efficacy in cancer. Front. Endocrinol. 9, 372. 10.3389/fendo.2018.00372 PMC6110930 30186229

[B5] Arneth B. Arneth R. Shams M. (2019). Metabolomics of type 1 and type 2 diabetes. Int. J. Mol. Sci. 20, 2467. 10.3390/ijms20102467 PMC6566263 31109071

[B6] Bagheri M. Farzadfar F. Qi L. Yekaninejad M. S. Chamari M. Zeleznik O. A. (2018). Obesity-related metabolomic profiles and discrimination of metabolically unhealthy obesity. J. Proteome Res. 17, 1452–1462. 10.1021/acs.jproteome.7b00802 29493238

[B7] Brandmaier S. Xu T. Illig T. Suhre K. Adamski J. Wang-Sattler R. (2015). Response to Comment on Xu et al. Effects of Metformin on Metabolite Profiles and LDL Cholesterol in Patients With Type 2 Diabetes. Diabetes Care 2015;38:1858-1867. Diabetes Care 38, e216–7. 10.2337/dci15-0022 26604292

[B8] Chatterjee S. Khunti K. Davies M. J. (2017). Type 2 diabetes. Lancet 389, 2239–2251. 10.1016/S0140-6736(17)30058-2 28190580

[B9] Chaudhury A. Duvoor C. Reddy Dendi V. S. Kraleti S. Chada A. Ravilla R. (2017). Clinical review of antidiabetic drugs: implications for type 2 diabetes mellitus management. Front. Endocrinol. 8, 6. 10.3389/fendo.2017.00006 PMC5256065 28167928

[B10] Chen S. Akter S. Kuwahara K. Matsushita Y. Nakagawa T. Konishi M. (2019). Serum amino acid profiles and risk of type 2 diabetes among Japanese adults in the hitachi health study. Sci. Rep. 9, 7010. 10.1038/s41598-019-43431-z 31065046 PMC6504928

[B11] Chen T. Ni Y. Ma X. Bao Y. Liu J. Huang F. (2016). Branched-chain and aromatic amino acid profiles and diabetes risk in Chinese populations. Sci. Rep. 6, 20594. 10.1038/srep20594 26846565 PMC4742847

[B12] Cheng S. Rhee E. P. Larson M. G. Lewis G. D. McCabe E. L. Shen D. (2012). Metabolite profiling identifies pathways associated with metabolic risk in humans. Circulation 125, 2222–2231. 10.1161/circulationaha.111.067827 22496159 PMC3376658

[B13] Dahabiyeh L. A. Malkawi A. K. Wang X. Colak D. Mujamammi A. H. Sabi E. M. (2020). Dexamethasone-induced perturbations in tissue metabolomics revealed by chemical isotope labeling LC-MS analysis. Metabolites 10, 42. 10.3390/metabo10020042 PMC7074358 31973046

[B14] Diabetes Prevention Program Research Group (2012). Long-term safety, tolerability, and weight loss associated with metformin in the diabetes prevention program outcomes study. Diabetes Care 35, 731–737. 10.2337/dc11-1299 22442396 PMC3308305

[B15] Farriol M. Segovia-Silvestre T. Castellanos J. M. Venereo Y. Orta X. (2001). Role of putrescine in cell proliferation in a colon carcinoma cell line. Nutrition 17, 934–938. 10.1016/s0899-9007(01)00670-0 11744344

[B16] Ferrannini E. Natali A. Camastra S. Nannipieri M. Mari A. Adam K. P. (2013). Early metabolic markers of the development of dysglycemia and type 2 diabetes and their physiological significance. Diabetes 62, 1730. 10.2337/db12-0707 23160532 PMC3636608

[B17] Floegel A. Stefan N. Yu Z. Mühlenbruch K. Drogan D. Joost H. G. (2013a). Identification of serum metabolites associated with risk of type 2 diabetes using a targeted metabolomic approach. Diabetes 62, 639. 10.2337/db12-0495 23043162 PMC3554384

[B18] Floegel A. Stefan N. Yu Z. Mühlenbruch K. Drogan D. Joost H. G. (2013b). Identification of serum metabolites associated with risk of type 2 diabetes using a targeted metabolomic approach. Diabetes 62, 639–648. 10.2337/db12-0495 23043162 PMC3554384

[B19] Foretz M. Guigas B. Viollet B. (2019). Understanding the glucoregulatory mechanisms of metformin in type 2 diabetes mellitus. Nat. Rev. Endocrinol. 15, 569–589. 10.1038/s41574-019-0242-2 31439934

[B20] Fried M. Yumuk V. Oppert J. M. Scopinaro N. Torres A. J. Weiner R. (2013). Interdisciplinary European Guidelines on metabolic and bariatric surgery. Obes. Facts 6, 449–468. 10.1159/000355480 24135948 PMC5644681

[B21] Goyal R. J. I. (2020). Diabetes mellitus type 2. Available at: https://www.ncbi.nlm.nih.gov/books/NBK513253/.*StatPearls* from.

[B101] Gu X. Al Dubayee M. Alshahrani A. Masood A. Benabdelkamel H. Zahra M. (2020). Distinctive metabolomics patterns associated with insulin resistance and type 2 diabetes mellitus. Front. Mol. Biosci. 7, 609806. 10.3389/fmolb.2020.609806 33381523 PMC7768025

[B22] Guasch-Ferré M. Hruby A. Toledo E. Clish C. B. Martínez-González M. A. Salas-Salvadó J. (2016). Metabolomics in prediabetes and diabetes: a systematic review and meta-analysis. Diabetes Care 39, 833. 10.2337/dc15-2251 27208380 PMC4839172

[B23] Ha C. Y. Kim J. Y. Paik J. K. Kim O. Y. Paik Y. H. Lee E. J. (2012). The association of specific metabolites of lipid metabolism with markers of oxidative stress, inflammation and arterial stiffness in men with newly diagnosed type 2 diabetes. Clin. Endocrinol. 76, 674–682. 10.1111/j.1365-2265.2011.04244.x 21958081

[B24] Haufroid M. Wouters J. (2019). Targeting the serine pathway: a promising approach against tuberculosis? Pharmaceuticals 12, 66. 10.3390/ph12020066 PMC6630544 31052291

[B25] Ho J. E. Larson M. G. Ghorbani A. Cheng S. Chen M. H. Keyes M. (2016). Metabolomic profiles of body mass index in the framingham heart study reveal distinct cardiometabolic phenotypes. PloS One 11, e0148361. 10.1371/journal.pone.0148361 26863521 PMC4749349

[B26] Huan T. Li L. (2015). Counting missing values in a metabolite-intensity data set for measuring the analytical performance of a metabolomics platform. Anal. Chem. 87, 1306–1313. 10.1021/ac5039994 25496403

[B27] Huan T. Wu Y. Tang C. Lin G. Li L. (2015). DnsID in MyCompoundID for rapid identification of dansylated amine- and phenol-containing metabolites in LC-MS-based metabolomics. Anal. Chem. 87, 9838–9845. 10.1021/acs.analchem.5b02282 26327437

[B28] Irving B. A. Carter R. E. Soop M. Weymiller A. Syed H. Karakelides H. (2015). Effect of insulin sensitizer therapy on amino acids and their metabolites. Metab. Clin. Exp. 64, 720–728. 10.1016/j.metabol.2015.01.008 25733201 PMC4525767

[B29] Jacob M. Gu X. Luo X. Al-Mousa H. Arnaout R. Al-Saud B. (2019a). Metabolomics distinguishes DOCK8 deficiency from atopic dermatitis: towards a biomarker discovery. Metabolites 9, 274. 10.3390/metabo9110274 PMC6918408 31718082

[B30] Jacob M. Lopata A. L. Dasouki M. Abdel Rahman A. M. (2019b). Metabolomics toward personalized medicine. Mass Spectrom. Rev. 38, 221–238. 10.1002/mas.21548 29073341

[B31] Jacob M. Malkawi A. Albast N. Al Bougha S. Lopata A. Dasouki M. (2018). A targeted metabolomics approach for clinical diagnosis of inborn errors of metabolism. Anal. Chim. Acta 1025, 141–153. 10.1016/j.aca.2018.03.058 29801603

[B32] Kahn S. E. Hull R. L. Utzschneider K. M. (2006). Mechanisms linking obesity to insulin resistance and type 2 diabetes. Nature 444, 840–846. 10.1038/nature05482 17167471

[B33] Kurland I. J. Accili D. Burant C. Fischer S. M. Kahn B. B. Newgard C. B. (2013). Application of combined omics platforms to accelerate biomedical discovery in diabesity. Ann. N. Y. Acad. Sci. 1287, 1–16. 10.1111/nyas.12116 23659636 PMC3709136

[B34] Leitner D. R. Frühbeck G. Yumuk V. Schindler K. Micic D. Woodward E. (2017). Obesity and type 2 diabetes: two diseases with a need for combined treatment strategies–EASO can lead the way. Obes. Facts 10, 483–492. 10.1159/000480525 29020674 PMC5741209

[B35] Li L. Li R. Zhou J. Zuniga A. Stanislaus A. E. Wu Y. (2013). MyCompoundID: using an evidence-based metabolome library for metabolite identification. Anal. Chem. 85, 3401–3408. 10.1021/ac400099b 23373753

[B36] Libert D. M. Nowacki A. S. Natowicz M. R. (2018). Metabolomic analysis of obesity, metabolic syndrome, and type 2 diabetes: amino acid and acylcarnitine levels change along a spectrum of metabolic wellness. PeerJ 6, e5410. 10.7717/peerj.5410 30186675 PMC6120443

[B37] Liu X. Locasale J. W. (2017). Metabolomics: a primer. Trends Biochem. Sci. 42, 274–284. 10.1016/j.tibs.2017.01.004 28196646 PMC5376220

[B38] Ma Q. Li Y. Wang M. Tang Z. Wang T. Liu C. (2018). Progress in metabonomics of type 2 diabetes mellitus. Molecules 23, 1834. 10.3390/molecules23071834 PMC6100487 30041493

[B39] Madiraju A. K. Erion D. M. Rahimi Y. Zhang X. M. Braddock D. T. Albright R. A. (2014). Metformin suppresses gluconeogenesis by inhibiting mitochondrial glycerophosphate dehydrogenase. Nature 510, 542–546. 10.1038/nature13270 24847880 PMC4074244

[B40] Martin A. M. Young R. L. Leong L. Rogers G. B. Spencer N. J. Jessup C. F. (2017). The diverse metabolic roles of peripheral serotonin. Endocrinology 158, 1049–1063. 10.1210/en.2016-1839 28323941

[B41] Matsuoka K. Kato K. Takao T. Ogawa M. Ishii Y. Shimizu F. (2017). Concentrations of various tryptophan metabolites are higher in patients with diabetes mellitus than in healthy aged male adults. Diabetol Int. 8, 69–75. 10.1007/s13340-016-0282-y 30603309 PMC6224928

[B42] Menge B. A. Schrader H. Ritter P. R. Ellrichmann M. Uhl W. Schmidt W. E. (2010). Selective amino acid deficiency in patients with impaired glucose tolerance and type 2 diabetes. Regul. Pept. 160, 75–80. 10.1016/j.regpep.2009.08.001 19695292

[B43] Newgard C. B. An J. Bain J. R. Muehlbauer M. J. Stevens R. D. Lien L. F. (2009). A branched-chain amino acid-related metabolic signature that differentiates obese and lean humans and contributes to insulin resistance. Cell Metabol. 9, 311–326. 10.1016/j.cmet.2009.02.002 PMC3640280 19356713

[B44] Newgard C. B. (2012). Interplay between lipids and branched-chain amino acids in development of insulin resistance. Cell Metabol. 15, 606–614. 10.1016/j.cmet.2012.01.024 PMC3695706 22560213

[B45] Ostojic S. Vranes M. Loncar D. Zenic N. Sekulic D. (2018). Guanidinoacetic acid and creatine are associated with cardiometabolic risk factors in healthy men and women: a cross-sectional study. Nutrients 10, 87. 10.3390/nu10010087 PMC5793315 29342866

[B46] Park S. Sadanala K. C. Kim E. K. (2015). A metabolomic approach to understanding the metabolic link between obesity and diabetes. Mol. Cell. 38, 587–596. 10.14348/molcells.2015.0126 PMC4507023 26072981

[B47] Post A. Tsikas D. Bakker S. J. L. (2019). Creatine is a conditionally essential nutrient in chronic kidney disease: a hypothesis and narrative literature review. Nutrients 11, 1044. 10.3390/nu11051044 PMC6567063 31083291

[B48] Rangel-Huerta O. D. Pastor-Villaescusa B. Gil A. (2019). Are we close to defining a metabolomic signature of human obesity? A systematic review of metabolomics studies. Metabolomics 15, 93. 10.1007/s11306-019-1553-y 31197497 PMC6565659

[B49] Rena G. Hardie D. G. Pearson E. R. (2017). The mechanisms of action of metformin. Diabetologia 60, 1577–1585. 10.1007/s00125-017-4342-z 28776086 PMC5552828

[B50] Roberts L. D. Koulman A. Griffin J. L. (2014). Towards metabolic biomarkers of insulin resistance and type 2 diabetes: progress from the metabolome. Lancet Diabetes Endocrinol. 2, 65–75. 10.1016/s2213-8587(13)70143-8 24622670

[B51] Rotroff D. M. Oki N. O. Liang X. Yee S. W. Stocker S. L. Corum D. G. (2016). Pharmacometabolomic assessment of metformin in non-diabetic, african Americans. Front. Pharmacol. 7, 135. 10.3389/fphar.2016.00135 27378919 PMC4906013

[B52] Saladini S. Aventaggiato M. Barreca F. Morgante E. Sansone L. Russo M. A. (2019). Metformin impairs glutamine metabolism and autophagy in tumour cells. Cells 8, 49. 10.3390/cells8010049 PMC6356289 30646605

[B53] Tomasova P. Buganova M. Pelantova H. Holubova M. Sediva B. Zelezna B. (2019). Metabolomics based on MS in mice with diet-induced obesity and type 2 diabetes mellitus: the effect of vildagliptin, metformin, and their combination. Appl. Biochem. Biotechnol. 188, 165–184. 10.1007/s12010-018-2899-8 30393821

[B54] UK Prospective Diabetes Study Group (1998). Effect of intensive blood-glucose control with metformin on complications in overweight patients with type 2 diabetes (UKPDS 34). UK Prospective Diabetes Study (UKPDS) Group. Lancet 352, 854–865. 9742977

[B55] Urpi-Sarda M. Almanza-Aguilera E. Llorach R. Vázquez-Fresno R. Estruch R. Corella D. (2019). Non-targeted metabolomic biomarkers and metabotypes of type 2 diabetes: a cross-sectional study of PREDIMED trial participants. Diabetes Metab. 45, 167–174. 10.1016/j.diabet.2018.02.006 29555466

[B56] Verma S. Hussain M. E. (2017). Obesity and diabetes: an update. Diabetes Metab. Syndr. 11, 73–79. 10.1016/j.dsx.2016.06.017 27353549

[B57] Vial G. Detaille D. Guigas B. (2019). Role of mitochondria in the mechanism(s) of action of metformin. Front. Endocrinol. 10, 294. 10.3389/fendo.2019.00294 PMC6514102 31133988

[B58] Viollet B. Guigas B. Sanz Garcia N. Leclerc J. Foretz M. Andreelli F. (2012). Cellular and molecular mechanisms of metformin: an overview. Clin. Sci. 122, 253–270. 10.1042/cs20110386 PMC3398862 22117616

[B59] Walford G. A. Davis J. Warner A. S. Ackerman R. J. Billings L. K. Chamarthi B. (2013). Branched chain and aromatic amino acids change acutely following two medical therapies for type 2 diabetes mellitus. Metab. Clin. Exp. 62, 1772–1778. 10.1016/j.metabol.2013.07.003 23953891 PMC3833885

[B60] Wang T. J. Larson M. G. Vasan R. S. Cheng S. Rhee E. P. McCabe E. (2011). Metabolite profiles and the risk of developing diabetes. Nat. Med. 17, 448–453. 10.1038/nm.2307 21423183 PMC3126616

[B61] Wang-Sattler R. Yu Z. Herder C. Messias A. C. Floegel A. He Y. (2012). Novel biomarkers for pre-diabetes identified by metabolomics. Mol. Syst. Biol. 8, 615. 10.1038/msb.2012.43 23010998 PMC3472689

[B62] Wu Y. Li L. (2012). Determination of total concentration of chemically labeled metabolites as a means of metabolome sample normalization and sample loading optimization in mass spectrometry-based metabolomics. Anal. Chem. 84, 10723–10731. 10.1021/ac3025625 23190334

[B63] Yan M. Qi H. Xia T. Zhao X. Wang W. Wang Z. (2019). Metabolomics profiling of metformin-mediated metabolic reprogramming bypassing AMPKα. Metabolism 91, 18–29. 10.1016/j.metabol.2018.11.010 30468782

[B64] Yee S. W. Lin L. Merski M. Keiser M. J. Gupta A. Zhang Y. (2015). Prediction and validation of enzyme and transporter off-targets for metformin. J. Pharmacokinet. Pharmacodyn. 42, 463–475. 10.1007/s10928-015-9436-y 26335661 PMC4656030

[B65] Zhang A. Sun H. Wang X. (2017). Emerging role and recent applications of metabolomics biomarkers in obesity disease research. RSC Adv. 7, 14966–14973. 10.1039/c6ra28715h

[B66] Zheng Y. Ley S. H. Hu F. B. (2018). Global aetiology and epidemiology of type 2 diabetes mellitus and its complications. Nat. Rev. Endocrinol. 14, 88–98. 10.1038/nrendo.2017.151 29219149

[B67] Zhou R. Tseng C. L. Huan T. Li L. (2014). IsoMS: automated processing of LC-MS data generated by a chemical isotope labeling metabolomics platform. Anal. Chem. 86, 4675–4679. 10.1021/ac5009089 24766305

