# Supplementary figures and images for: Obesity Connected Metabolic Changes in Type 2 Diabetic Patients Treated With Metformin
Source: Front Pharmacol. 2021 Feb 16;11:616157. doi: 10.3389/fphar.2020.616157 (PMC7921791; doi:10.3389/fphar.2020.616157)

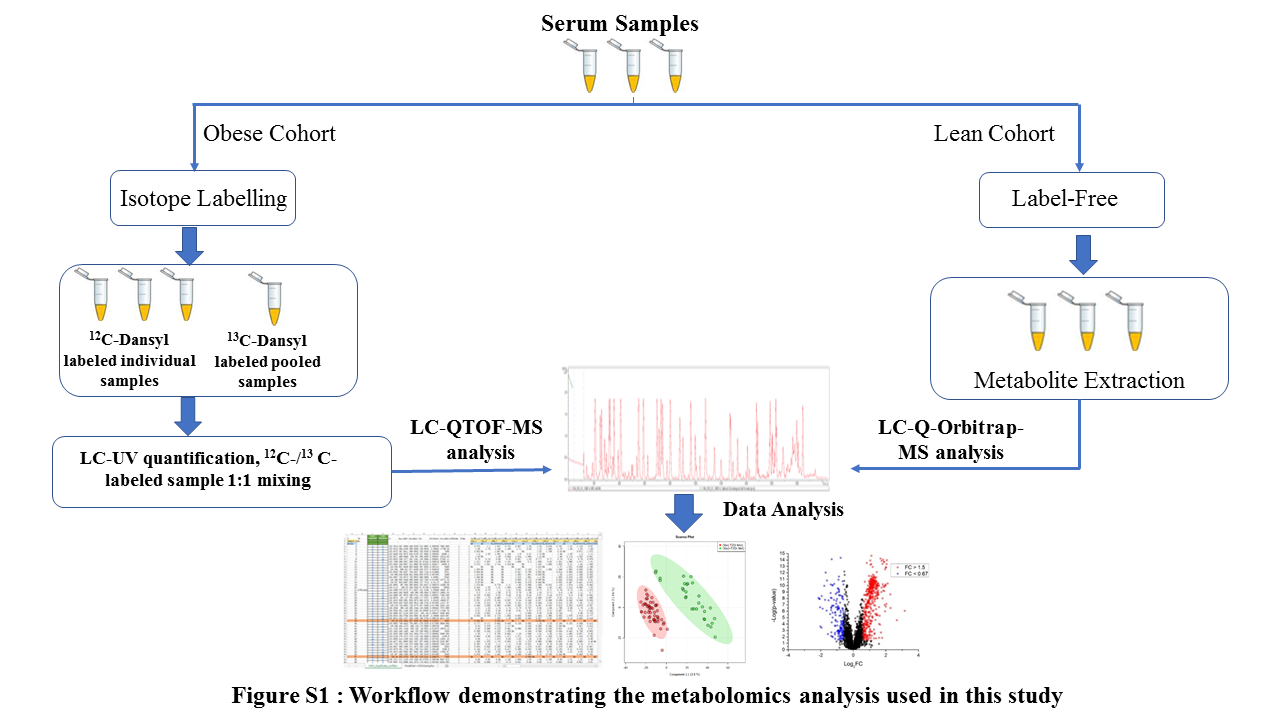

Supplement: Supplementary file 5 [file image1.tif]

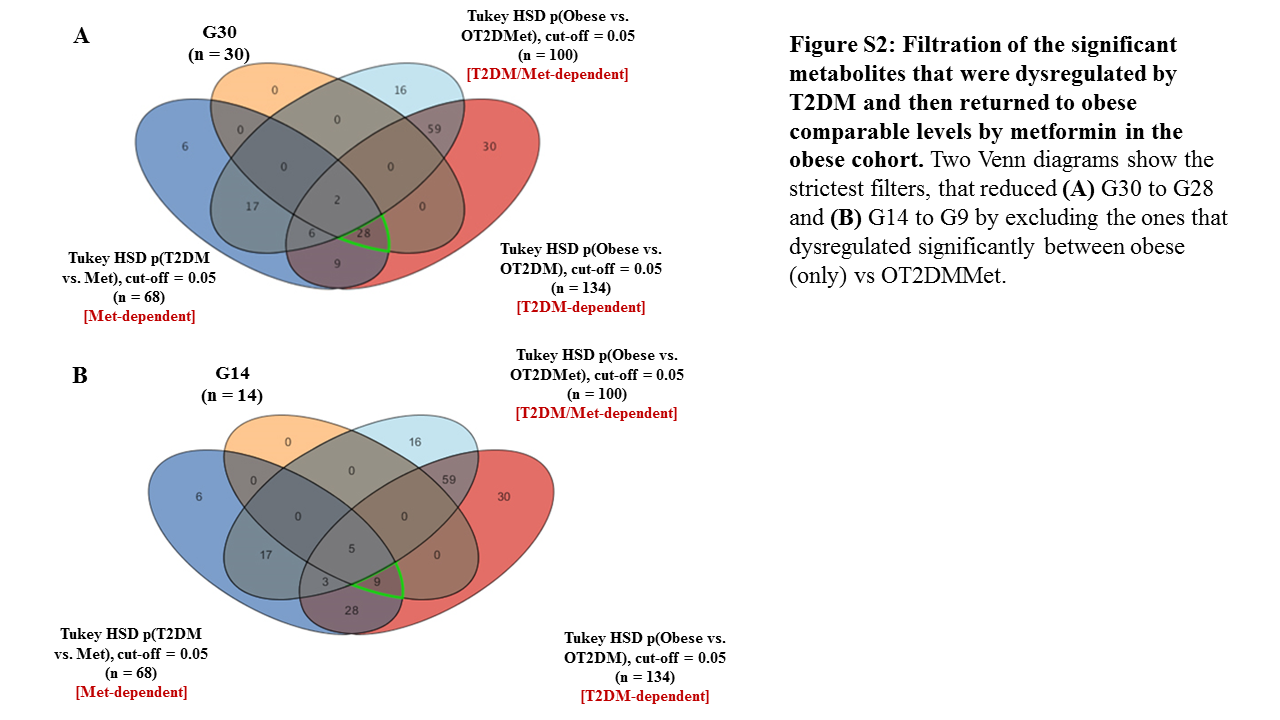

Supplement: Supplementary file 6 [file image2.tif]

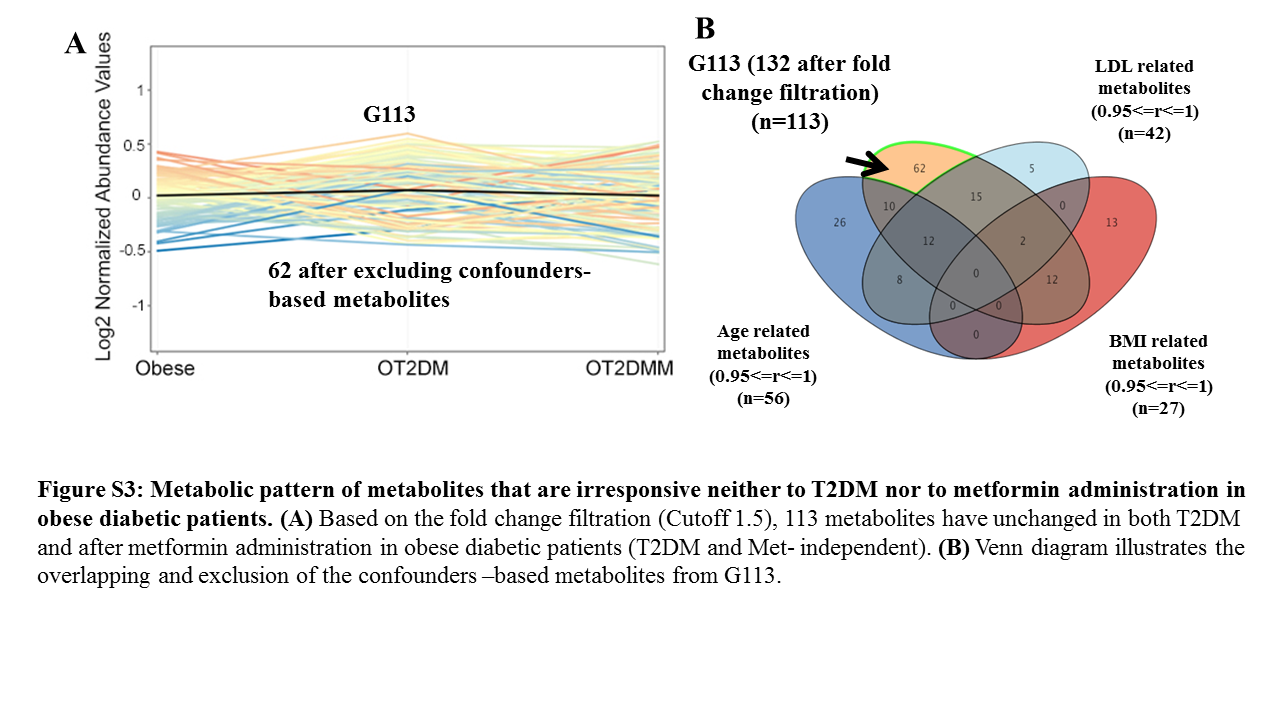

Supplement: Supplementary file 7 [file image3.tif]
